# Vick's Floral Guide for 1876

**Published:** 1876-01

**Authors:** 


*Vick's Floral Guide for* 1876.—This is a beautiful quarterly Journal, finely illustrated and an elegant colored frontispiece with the first number. The number before us for January, 1876, contains a beautiful frontispiece of annuals, and is a gem. Besides what relates to floral matter, this number contains an interesting account of a trip to the Pacific, with a good description of the monster trees and the Yosemite valley. The guide is published by the well known Florist, Mr. Jas. Vick, of Rochester, N. Y., at the low price merely nominal, of 25 cents a year, and will prove interesting and instructive to all who love flowers and vegetables.

We take pleasure in recommending the excellent seeds furnished by Mr. Vick, both flower and vegetable, which are planted by millions of people in America with the most successful results. Mr. Vick also publishes "The Flower and Vegetable Garden," the most beautiful book of the kind in the world. It contains nearly 150 pages, hundreds of fine illustrations and four chromo plates of flowers beautifully drawn and colored from nature. The price is 35 cents in paper, or 65 cents in elegant cloth.

A price catalogue of seeds, etc., is sent free to all who enclose the postage.

